# Structural analysis of the recognition of the -35 promoter element by SigW from *Bacillus subtilis*

**DOI:** 10.1371/journal.pone.0221666

**Published:** 2019-08-28

**Authors:** Eunju Kwon, Shankar Raj Devkota, Deepak Pathak, Pawan Dahal, Dong Young Kim

**Affiliations:** College of Pharmacy, Yeungnam University, Gyeongsan, Gyeongbuk, South Korea; Instituto de Biologia Molecular de Barcelona, SPAIN

## Abstract

Sigma factors are key proteins that mediate the recruitment of RNA polymerase to the promoter regions of genes, for the initiation of bacterial transcription. Multiple sigma factors in a bacterium selectively recognize their cognate promoter sequences, thereby inducing the expression of their own regulons. In this paper, we report the crystal structure of the σ4 domain of *Bacillus subtilis* SigW bound to the -35 promoter element. Purine-specific hydrogen bonds of the -35 promoter element with the recognition helix α9 of the σ4 domain occurs at three nucleotides of the consensus sequence (G_-35_, A_-34_, and G’_-31_ in G_-35_A_-34_A_-33_A_-32_C_-31_C_-30_T_-29_). The hydrogen bonds of the backbone with the α7 and α8 of the σ4 domain occurs at G’_-30_. These results elucidate the structural basis of the selective recognition of the promoter by SigW. In addition, comparison of SigW structures complexed with the -35 promoter element or with anti-sigma RsiW reveals that DNA recognition and anti-sigma factor binding of SigW are mutually exclusive.

## Introduction

Transcription in bacteria is initiated by sigma factors, which recruit the core RNA polymerase to a cognate promoter [1, 2]. Sigma factors selectively recognize promoter elements, -10 and -35 elements, and additional sequences, including extended -10 element and discriminator, which are present upstream of the transcription start site [3, 4]. During transcription initiation, the -10 element is strand-separated to form a transcription bubble [5, 6], whereas the -35 element is recognized by a helix-turn-helix (HTH) motif in the sigma factor, without strand separation [7, 8].

Sigma factors are categorized into two families, based on the sequence homology with *Escherichia coli* sigma factors: housekeeping σ^70^ required for bacterial homeostasis; and σ^54^ activated for nitrogen utilization [9]. The σ^70^ family is further sub-divided into five groups [4, 10]. Group I sigma factors are composed of σ_1.1_, σ_2_, σ_3_, and σ_4_ domains, which are responsible for the recognition of the discriminator, -10, extended -10, and -35 elements, respectively. The primary sigma factors, which regulate the transcription of housekeeping genes, belong to group I. Group II-V are classified depending on the presence or absence of the domains and motifs in group I. Alternative sigma factors, which are activated in response to a range of stress conditions, belong to groups II-V [10].

Group IV sigma factors are known as extracytoplasmic function (ECF) sigma factors, because they are activated in response to extracytoplasmic stresses [3, 10, 11]. They are the most divergent group of sigma factors, and almost all bacteria contain multiple ECF sigma factors. As an extreme case, *Streptomyces coelicolor* contains approximately 50 ECF sigma factors in its genome [10, 12]. The ECF sigma factors are composed of only σ_2_ and σ_4_ domains, which bind -10 and -35 elements, respectively [3, 13–15]. In many cases, promoter binding of ECF sigma factors is inhibited by binding to the cytoplasmic domain of transmembrane anti-sigma factors [10, 11, 16], and is activated to initiate the transcription of target genes in response to a specific stress signal by being released from the anti-sigma factor.

*Bacillus subtilis* contains at least seven ECF sigma factors: SigM, SigV, SigW, SigX, SigY, SigZ, and YlaC [17]. Of these sigma factors, SigW induces the transcription of its target regulon to counteract cell envelope stresses caused by antibiotics [18, 19], alkaline pH [20], and high salt concentration [21, 22]. In the absence of these stresses, SigW is downregulated by anti-sigma factor RsiW, which is localized in the plasma membrane [16, 23]. It is released from the anti-sigma factor under the stress conditions [24–27]. Under these conditions, transmembrane RsiW is sequentially cleaved by regulated intramembrane proteolysis by PrsW and RasP proteases; therefore, the cytoplasmic domain of RsiW is released together with SigW from the plasma membrane [25, 27]. Subsequently, the cytoplasmic domain of RsiW is completely degraded by ClpXP protease [28], allowing SigW to bind to the core RNA polymerase, and thereby to activate SigW-dependent transcription.

Even though crystal structures have been reported showing interactions between the σ_4_ domain and the -35 promoter element in group I and group IV sigma factors [3, 7, 8, 29, 30], structural information from diverse sigma factors is required to understand the selective recognition of the -35 element. In the work reported here, we determined the crystal structure of the σ^W^_4_/-35 element complex, and analyzed the promoter binding mode of SigW. The structure reveals the unique regulation mode of SigW-dependent transcription, and the recognition specificity of the -35 element by the σ_4_ domain.

## Materials and methods

### Plasmid preparation and protein expression

DNA encoding the σ4 domain of *B*. *subtilis* SigW (σ^W^_4_; residues 126–187) was amplified from the genome of *B*. *subtilis* 168 strain using polymerase chain reaction and inserted into pETDuet-1 vector (Merck Millipore, Billerica, MA, USA) expressing the N-terminal 6_X_His tag and TEV protease cleavage site. The plasmid was transformed into *E*. *coli* strain BL21-star (DE3) (Thermo Fischer Scientific, Waltham, MA, USA) and cells were grown in Luria Broth media at 37°C. When the culture reached an OD_600_ of 0.6–0.7, the medium was cooled, and 0.4 mM isopropyl β-D-1-thiogalactopyranoside was added to the culture to induce σ^W^_4_ expression. After overnight incubation at 15°C, the cells were harvested by centrifugation at 3,000 *g* for 10 min.

### Purification of σ^W^_4_/-35^W^

Cells expressing 6_X_His-σ^W^_4_ were resuspended in buffer A (20 mM HEPES pH 7.5, 1.0 M NaCl, and 10% (v/v) glycerol), lysed by sonication, and clarified by centrifugation at 20,000 g for 30 min, after the addition of DNase I and RNase A at a concentration of 10 μg/ml. σ^W^_4_ was purified by immobilized metal affinity chromatography (IMAC) and size exclusion chromatography (SEC). Clarified cell lysate was loaded onto a 5 mL HisTrap nickel chelating column (GE Healthcare Bio-sciences, Uppsala, Sweden), and the resin was washed with buffer A, containing 80 mM imidazole. Proteins bound to the resin were eluted by an imidazole gradient (0.08–1.00 M imidazole). Fractions that contained 6_X_His-σ^W^_4_ were pooled and treated with TEV protease overnight at 25°C to cleave the His tag. After complete cleavage, the protein solution was dialyzed against buffer A for 3 h and passed through Ni-NTA resin to remove the 6_X_His tag (Thermo Fisher Scientific, Rockford, IL, USA). σ^W^_4_ was further purified by SEC using Superdex 75 preparatory grade column (GE Healthcare Biosciences) pre-equilibrated with buffer B (20 mM HEPES pH 7.5, 1.0 M NaCl, and 5% (v/v) glycerol).

The -35 element recognized by σ^W^_4_ was prepared by mixing two complementary DNA fragments. Two single-stranded DNAs (5’-ATTGAAACCTTT-3’ and 5’-AAAAGGTTTCAA-3’) were synthesized (Biobasic, Seoul, South Korea), mixed at a 1:1 molar ratio in buffer B, and purified by SEC using a Superdex75 analytical column. The purified -35 element (-35^W^) was then mixed with σ^W^_4_ at a 1.1:1 molar ratio. The mixture was dialyzed in buffer C (20 mM HEPES pH 7.5, 0.2 M NaCl, and 5% (v/v) glycerol) and concentrated to 15 mg/mL for crystal screening.

### Crystallization, data collection, and structure determination

Crystallization of the σ^W^_4_/-35^W^ complex was performed using the micro-batch method at 20°C. The drop for crystal screening was prepared by mixing 1 μL of σ^W^_4_/-35^W^ (15 mg/mL) and 1 μL of crystallization solution under a layer of Al’s oil (Hampton Research, Aliso Viejo, Ca, USA). Crystals of σ^W^_4_/-35^W^ grew completely in a month under the conditions of Wizard Precipitant Synergy 127 (0.1M imidazole/hydrochloric acid pH 6.5, 30% (v/v) PEG1500, 10% (v/v) isopropanol, and 0.1 M CaCl_2_) (Rigaku, Tokyo, Japan). Crystals of σ^W^_4_/-35^W^ were picked using a cryo-loop (Hampton Research) and flash-frozen in a cold nitrogen stream. Diffraction data were collected at PLS-BL7A (Beam line 7A, Pohang Light Source, South Korea) [31] and were indexed, integrated, and scaled using MOSFLM [32].

The crystal structure of σ^W^_4_/-35^W^ was determined by the molecular replacement (MR) method using PHASER [33]. The structure of the *E*. *coli* σ^E^_4_/-35 element (PDB ID: 2H27) was used as a template for MR. MR solution was found from the truncated σ^E^_4_ (residues 127–186)/-35 element. Cycles of refinement and model building were performed at 3.1 Å resolution using PHENIX.refine [34] and COOT [35]. Final refinement resulted in R / R_free_ values of 24.8 / 29.0% without residues in the disallowed region of the Ramachandran plot. The data collection and refinement statistics are summarized in Table 1. The final coordinates and structure factors were deposited in the Protein Data Bank (PDB ID: 6JHE). Structural alignment was performed using the DALI server [36]. Protein-ligand interactions were analyzed with LigPlot+ [37] and PDBePISA [38]. The free energy change (Δ*G*) of the protein caused by ligand binding was analyzed using PDBePISA [38]. The conversion of Δ*G* to a dissociation constant (*K*d) was calculated using the equation *K*d = e^(Δ*G*/RT*)*^ (R = 1.987 cal/molK; T = 293K). DNA geometry was analyzed using w3DNA [39]. Surface charge distribution was calculated using APBS [40]. The figures were drawn using PyMOL [41] and ALSCRIPT [42].

**Table 1:** Data collection and refinement statistics.

**Data collection**		
Data set		σ^W^_4_/-35^W^
Space group		P6_5_22
Unit cell		
	a, b, c (Å)	61.61, 61.61, 119.96
	α, β, γ (°)	90.00, 90.00, 90.00
Resolution (Å)		30.0–3.00 (3.18–3.00)
Wavelength (Å)		0.97933
Total/Unique reflections		45479/3013
Completeness (%)		99.4 (100.0)
I/σ		62.8 (16.4)
R_merge_ (%)		10.4 (52.4)
**Refinement**		
Resolution		30.0–3.10
No. reflections, working/free		2715/140
R_work_/R_free_ (%)		24.8/29.0
No. atoms		884
	Protein	436
	DNA	448
B factors		69.0
RMSD		
	Bond length (Å)	0.012
	Bond angle (°)	1.438
Ramachandran plot (%)		
	Favor	94.1
	Allowed	5.9
	Disallowed	0.0

### Accession number

The final coordinates and structure factors were deposited in the Protein Data Bank (PDB ID: **6JHE** for σ^W^_4_/-35^W^).

## Results and discussion

### Overall structure

**Fig 1:**
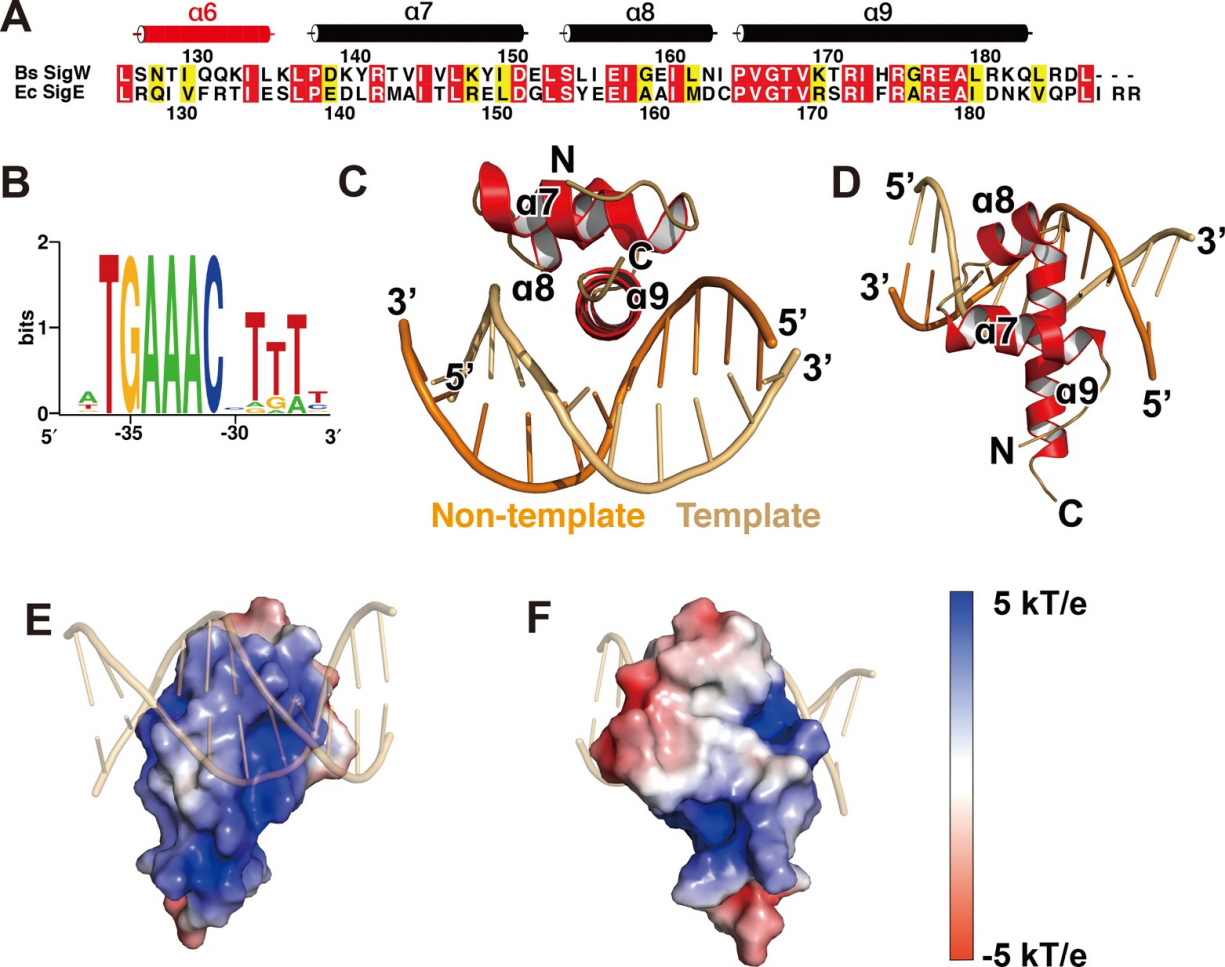
Structure of the σ^W^_4_/-35^W^ complex. (A) Sequence alignment of σ^W^_4_ and σ^E^_4_. The secondary structure of the σ^W^_4_ domain is displayed using a tube to indicate an α-helix. Identical residues are boxed in red, and similar residues are boxed in yellow. α6 disordered in the structure is displayed as a red tube, based on the crystal structure of the SigW/RsiW complex (PDB ID: 5WUQ). (B) Sequence logo for the -35 promoter element of the SigW regulon [44]. (C, D) Ribbon models of the σ^W^_4_/-35^W^ complex, drawn at two different orientations. α9 of σ^W^_4_ is inserted into the major groove of -35^W^ as a recognition helix of the HTH motif. (E, F) Surface model of σ^W^_4_ with charge distribution. The electrostatic potential, from red (-5 kT/e) to blue (+5 kT/e), is plotted on the solvent-accessible surface calculated with a solvent probe radius of 1.4 Å.

*B*. *subtilis* σ^W^_4_ (residues 125–187) recognizes a cognate -35 promoter element (**Fig 1A and 1B**). Its consensus sequence is identified as T_-36_G_-35_A_-34_A_-33_A_-32_C_-31_X_-30_T_-29_T_-28_T_-27_, based on the promoter sequences of the SigW regulon [43]. σ^W^_4_ was purified under high salt conditions (1 M NaCl) to minimize its instability, and dialyzed in low salt buffer together with the double-stranded DNA of A_-38_T_-37_T_-36_G_-35_A_-34_A_-33_A_-32_C_-31_C_-30_T_-29_T_-28_T_-27_ (-35^W^) to allow σ^W^_4_ binding to -35^W^. Crystals of σ^W^_4_/-35^W^ belonging to a hexagonal space group grew under conditions containing PEG1500 and isopropanol as protein precipitants. Diffraction data were collected at a resolution of 3.1 Å (**Table 1**), and the structure was determined by molecular replacement using the structure of a truncated *E*. *coli* σ^E^_4_/-35^E^ (-35 promoter element for SigE binding) as a template [8].

The crystal structure contains a σ^W^_4_ monomer and a double-stranded -35^W^ in the asymmetric unit. Residues 134–186 of SigW and 11 nucleotide pairs of -35^W^ were traced into the electron density (**S1 Fig**) and the final structure was refined at R / R_free_ values of 24.8 / 29.0% (Table 1). σ^W^_4_ is comprised of four α-helices (α6–α9) in the crystal structure of the SigW/RsiW complex [23]. However, residues 125–133, which correspond to α6, are disordered in the crystal structure of σ^W^_4_/-35^W^ (**Fig 1A**). The residues on α6 are likely to be flexible because they are not bound to DNA directly. α8–α9 of σ^W^_4_ forms the HTH motif, and α9 is inserted into the major groove of -35^W^ as a DNA recognition helix (**Fig 1C and 1D**) [7]. Positively-charged residues are distributed on the DNA binding surface of α8–α9, whereas hydrophobic patches are distributed on the opposite side that interacts with σ^W^_2_ in the crystal structure of SigW/RsiW (**Fig 1E and 1F**) [23].

### Interactions between σ^W^_4_ and the -35 promoter element

**Fig 2:**
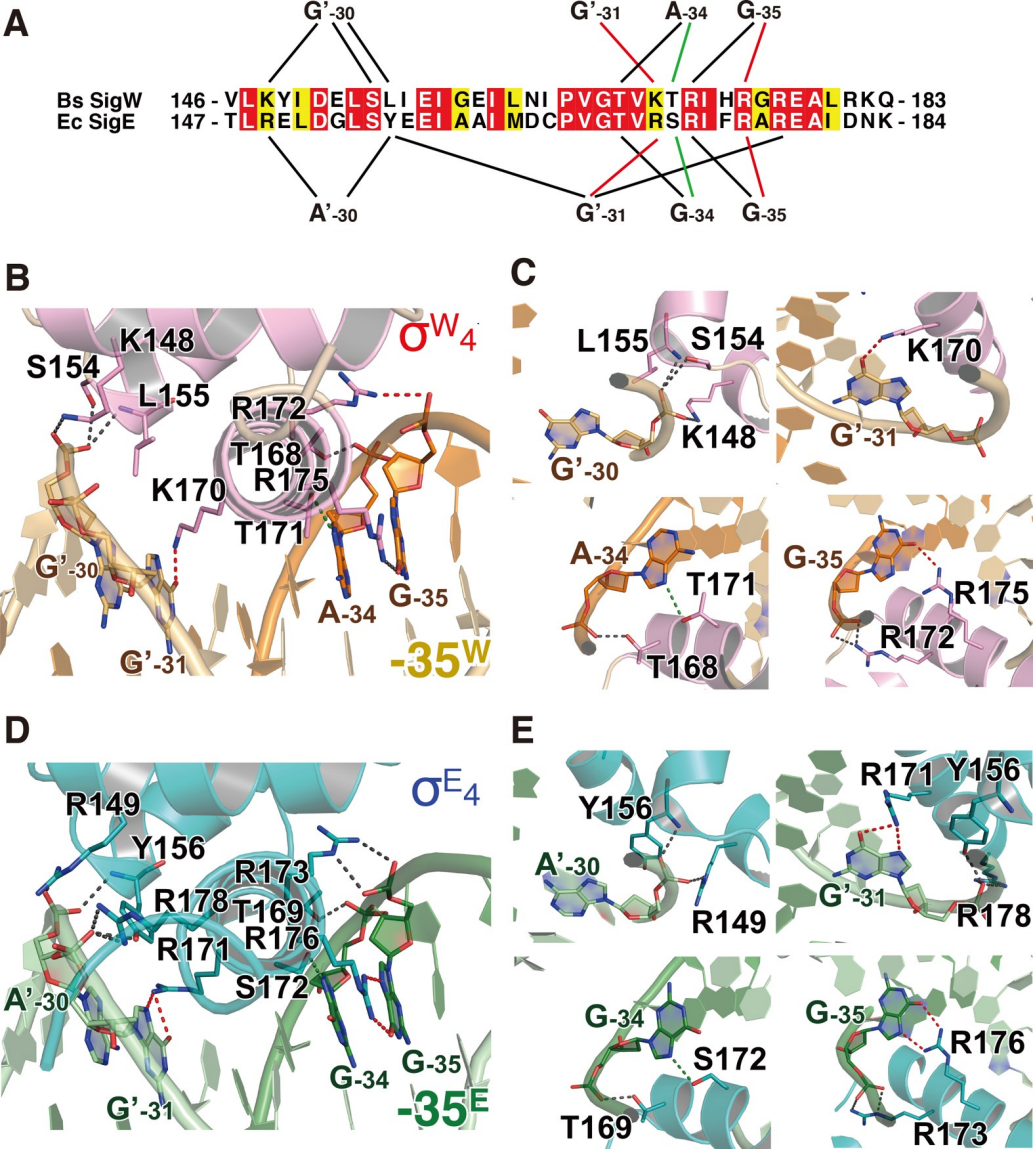
Interactions between σ_4_ domain and -35 promoter element. (A) Schematic diagram representing the hydrogen bonds between σ_4_ and the -35 element. The sequences of the -35 element-binding interface in *B*. *subtilis* SigW and *E*. *coli* SigE are aligned. Black lines indicate the backbone interactions, green lines show the purine-specific interactions, and red the guanine-specific interactions. (B, C) Hydrogen bonds between σ^W^_4_ and -35^W^. Residues and nucleotides, which form hydrogen bonds, are drawn as pink and orange stick models, respectively. Dotted lines indicate hydrogen bonds between σ^W^_4_ and -35^W^. (D, E) Interactions between σ^E^_4_ and -35^E^ (PDB ID: 2H27) [8]. Residues and nucleotides, which form hydrogen bonds, are drawn as teal and dark green stick models. The ribbon models are drawn at the same orientations as those in (B) and (C). Dotted lines indicate hydrogen bonds between σ^E^_4_ and -35^E^. The dotted lines in (B-E) are colored by the same scheme as the lines in (A).

The recognition helix α9 mediates the major interactions between σ^W^_4_ and -35^W^ through hydrogen bonds and hydrophobic interactions. The bases and backbones of three purine nucleotides (G_-35_ and A_-34_ in the non-template strand and G’_-31’_ in the template strand; ‘ indicates the template strand of DNA) form hydrogen bonds with the residues on the N-terminal half of α9 in σ^W^_4_ (**Fig 2A–2C and S2 Fig**). The guanine oxygen (O6) and backbone phosphate (OP2) of G_-35_ form hydrogen bonds with side chain amino groups (NH2) of R175 and R172, respectively. The purine nitrogen (N7) and backbone phosphate (OP2) of A_-34_ interact with the side chain oxygens (OG1) of T171 and T168. The guanine oxygen (O6) of G’_-31_ forms a hydrogen bond with the side chain amino group (NZ) of K170. The electron density map for the side chain of K170 is relatively weak; however, the most-preferred rotamer is at hydrogen bond distance to the O6 of G’_-31_ (**S2B Fig**). Hydrophobic interactions are observed between K170-G’_-31_T’_-32_, T171-A_-34_, H174-T’_-32_T’_-33_, and R175-T_-36_ (**S3 Fig**).

In addition to α9, α7-α8 contributes to -35^W^ binding without base specificity. The backbone atoms (OP1, OP2, and OP2) of G’_-30_ form hydrogen bonds with the side chain amino group (NZ) of K148, the side chain oxygen (OG) of S154, and the backbone nitrogen of L155, respectively (**Fig 2B and 2C**). Altogether, α9 in σ^W^_4_ specifically recognizes -35^W^, and α7-α8 provides additional contacts, leading to a tighter interaction.

### Structural comparison of σ^W^_4_/-35^W^ and σ^E^_4_/-35^E^

SigE is an *E*. *coli* ECF sigma factor activated in response to envelope stress and induces transcription of heat shock proteins [45]. Its σ_4_ domain (σ^E^_4_) recognizes the -35 element, of which the consensus sequence is G_-35_G_-34_A_-33_A_-32_C_-31_T_-30_T_-29_ (-35^E^) [46]. The σ^E^_4_ structure is highly similar to σ^W^_4_ [8]. σ^W^_4_ is superimposed on σ^E^_4_ with a root mean square deviation (RMSD) value of 1.8 Å for 53 Cα atoms (**S4A Fig**). The overall fold is conserved between σ^W^_4_ and σ^E^_4_ and the main differences are observed at the N- and C-termini (**S4A and S4B Fig**).

Overall, the interactions of σ^E^_4_ and σ^W^_4_ with the corresponding -35 elements are conserved. Three nucleotides, G_-35_, G_-34_, and G’_-31_, in -35^E^, which correspond to G_-35_, A_-34_, and G’_-31_ in -35^W^, mediate purine nucleotide-specific interactions with the recognition α-helix (**Fig 2A**). The backbone and base of G_-35_ (G_-35_ in σ^W^_4_) form hydrogen bonds with R173 (R172 in σ^W^_4_) and R176 (R175 in σ^W^_4_). The backbone and base of G_-34_ (A_-34_ in -35^W^) form hydrogen bonds with T169 (T168 in σ^W^_4_) and S172 (T171 in σ^W^_4_). Although the -34 position is not identical between -35^E^ and -35^W^ (G_-34_ in -35^E^ and A_-34_ in -35^W^), hydrogen bonds mediated by the backbone phosphate and purine N7 are conserved. The base of G’_-31_ forms a hydrogen bond with R171 (R170 in σ^W^_4_). The backbone phosphate of G’_-31_ also forms a hydrogen bond with R178, which is not observed in the σ^W^_4_/-35^W^ structure. A’_-30_ in-35^E^ mediates backbone interactions similarly to G’_-30_ in -35^W^ (**Fig 2**). The backbone phosphate of A’_-30_ forms hydrogen bonds with the NH1 of R149 (K148 in σ^W^_4_) and the backbone nitrogen of Y156 (L155 in σ^W^_4_) (**Fig 2D and 2E**). Interaction between S154 and A’_-30_ in σ^W^_4_/-35^W^ is missing in the σ^E^_4_/-35^E^ structure. In summary, purine base-specific hydrogen bonds in the structures of σ^W^_4_/-35^W^ and σ^E^_4_/-35^E^ are conserved, whereas the hydrogen bonds with the nucleotide backbone are slightly different.

Hydrophobic interactions between σ^E^_4_ and -35^E^ are mostly conserved in the σ^W^_4_/-35^W^ structure (**S3 Fig**). Residues R171, F175, and R176 in σ^E^_4_ (K170, H174, and R175 in σ^W^_4_) interact with T’_-32_, T’_-33_, and C_-36_ in -35^E^ (T’_-32_, T’_-33_, and T_-36_ in -35^W^), respectively. Hydrophobic interactions between P166/G168/T169 and G_-34_ and between Y156 and A’_-30_ are observed only in σ^E^_4_/-35^E^, whereas the interaction between K170 and G’_-31_ in σ^W^_4_/-35^W^ is observed only in σ^E^_4_/-35^E^. A cation-π interaction is observed between R176 and the pyrimidine ring of C’_-34_ in the structure of σ^E^_4_/-35^E^. However, the distance between the base (T_-36_) and the corresponding residue (R175) is too far to form a cation-π interaction in the structure of σ^W^_4_/-35^W^ (**S5 Fig**).

### DNA geometry of -35^W^

**Fig 3:**
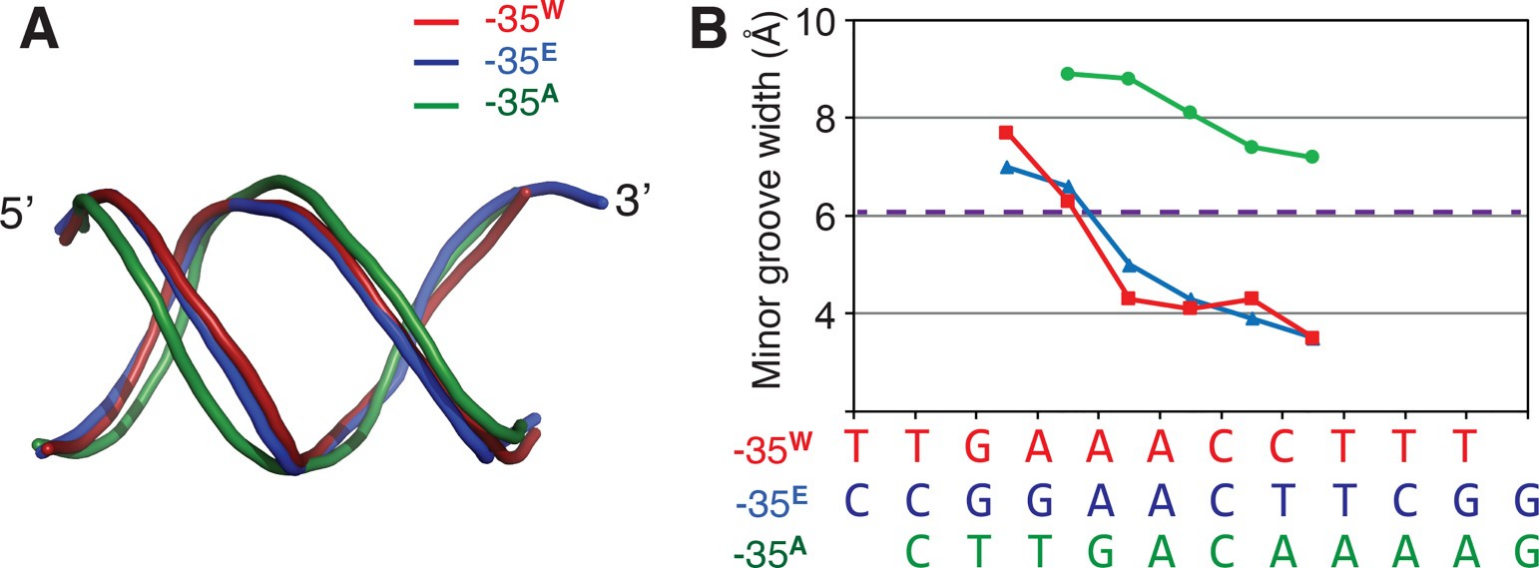
DNA conformation of the -35 promoter element. (A) DNA backbone geometry. -35 elements from the structures of σ^W^_4_/-35^W^, *E*.*coli* σ^E^_4_/-35^E^ (PDB ID: 2H27), and *Thermus aquaticus* σ^A^_4_/-35^A^ (PDB ID: 1KU7) are superposed. The 5′ and 3′-ends of the non-template strands are labeled. -35^W^, 35^E^, and -35^A^ are colored red, blue, and green, respectively. (B) Plot showing the minor groove width of the -35 elements. The sequences of the -35 elements are aligned with the plot. -35^W^ and -35^E^ have narrower minor grooves than a normal B-DNA, while -35^A^ contains a wider minor groove. The dashed purple line indicates the minor groove width of standard B-form DNA.

Nucleotides A_-33_A_-32_ in -35^E^ (G_-35_G_-34_A_-33_A_-32_C_-31_T_-30_T_-29_) do not form hydrogen bonds with σ^E^_4_, although these nucleotides are conserved among the -35 elements of the SigE regulon, and mutating these nucleotides in the *Salmonella enterica serovar Typhimurium* SigE has been shown to lead to defective transcription [47]. These nucleotides are involved in characteristic oligo(dA)/oligo(dT)-like DNA geometry that is rigid and straight with a narrow minor groove [8, 48]. A previous structural study of the σ^E^_4_/-35^E^ complex suggested that the geometry of the narrowed minor groove is critical for σ^E^_4_ recognition [8], and we show that -35^W^ also displays a narrowed minor groove (**Fig 3**). Like -35^E^, the narrowing of the minor groove of -35^W^ begins at A_-33_A_-32_ and is stabilized downstream of the -35 element, even though the downstream sequence of -35^W^ has an insertion of two cytosines (A_-33_A_-32_C_-31_C_-30_T_-29_) (**Fig 3**). In contrast, -G_-33_A_-32_ in -35^A^ (the -35 element of *Thermus aquaticus* SigA) has a wider minor groove than normal B-DNA (**Fig 3**). The crystal structure of σ^W^_4_/-35^W^ supports the suggestion that the A_-33_A_-32_ conservation in the -35 element for group IV sigma factors is critical for the formation of the narrow minor groove [8].

### Structural comparison of σ^W^_4_/-35^W^ and SigW/RsiW

**Fig 4:**
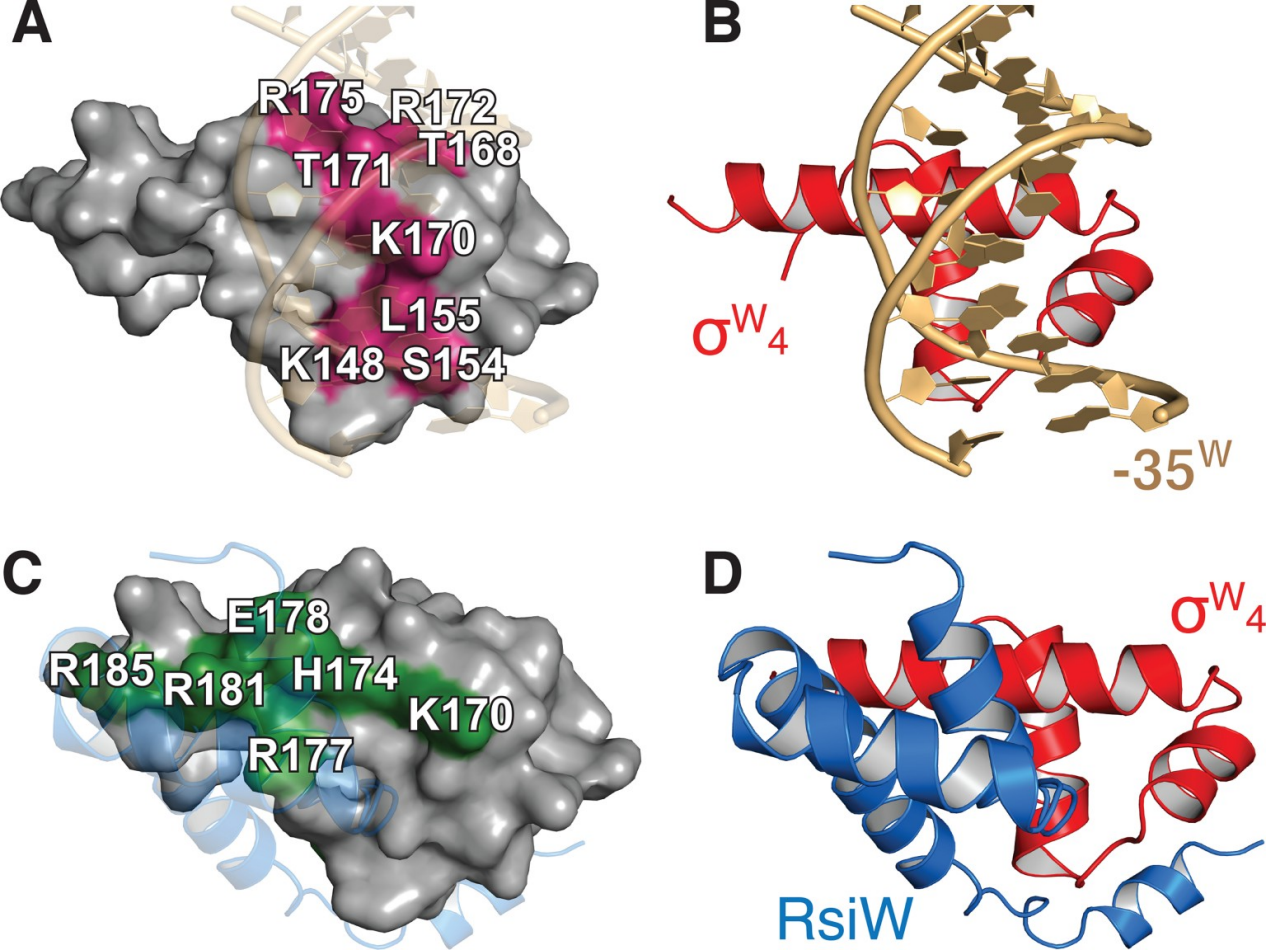
Structural comparison of σ^W^_4_/-35^W^ and SigW/RsiW. (A) Surface model of σ^W^_4_ bound to -35^W^. The magenta surface indicates the -35^W^ binding interface. Residues on the binding surface are labeled. (B) Ribbon model of σ^W^_4_/-35^W^ structure. (C) Surface model of σ^W^_4_ bound to RsiW. The RsiW binding surface is colored green, and the residues are labeled. (D) Ribbon model of σ^W^_4_ bound to RsiW. σ^W^_4_ structures in (A)-(D) are drawn at the same orientation.

The crystal structure of SigW complexed with anti-sigma RsiW was previously reported [23]. The structures of σ^W^_4_ under the binding of -35^W^ and RsiW superimpose with an RMSD value of 1.6 Å for 53 Cα atoms (**S4B Fig**). Although conformational differences of σ^W^_4_ in the structures bound to -35W and RsiW are minor, σ^W^_4_ bound to -35^W^ exists in a slightly compact conformation. σ^W^_4_ interacts with -35^W^ through the residues K148, S154, L155, T168, K170, T171, R172, and R175, with a surface area of 621.2 Å^2^ buried at the binding interface (**Fig 4A and 4B**). σ^W^_4_ interacts with RsiW through the residues I150, K170, H174, R177, E178, R181, R185, and L187, these interactions result in an approximately 50% larger burial of surface area (915.2 Å^2^) (**Fig 4C and 4D**). The surface area of σ^W^_4_ that binds -35^W^ and RsiW partially overlaps with residue K170 on σ^W^_4_ (**Fig 4A and 4C**), indicating that the interactions of -35^W^ and RsiW with σ^W^_4_ are mutually exclusive.

In the crystal structure of the SigW/RsiW complex, the -10 element-binding surface of σ^W^_2_ is buried in the surface of σ^W^_4_ [23], whereas the -35 element-binding surface of σ^W^_4_ is directly blocked by RsiW (**Fig 4**), suggesting that SigW inhibition by RsiW competes with -35 element binding in the promoter. The binding of the -35 element to σ^W^_4_ results in a Δ*G* of -10.5 kcal/mol, which corresponds to a *K*d value of 1.47 x 10^−8^ M. RsiW binding to SigW results in a surface burial of 1619.6 Å^2^, and a Δ*G* of -17.85 kcal/mol. The free energy change corresponds to a *K*d value of 4.84 x 10^−14^ M. These observations indicate that DNA binding of SigW is structurally repressed in the presence of RsiW.

### Role of conserved residues in σ^W^_4_

**Fig 5:**
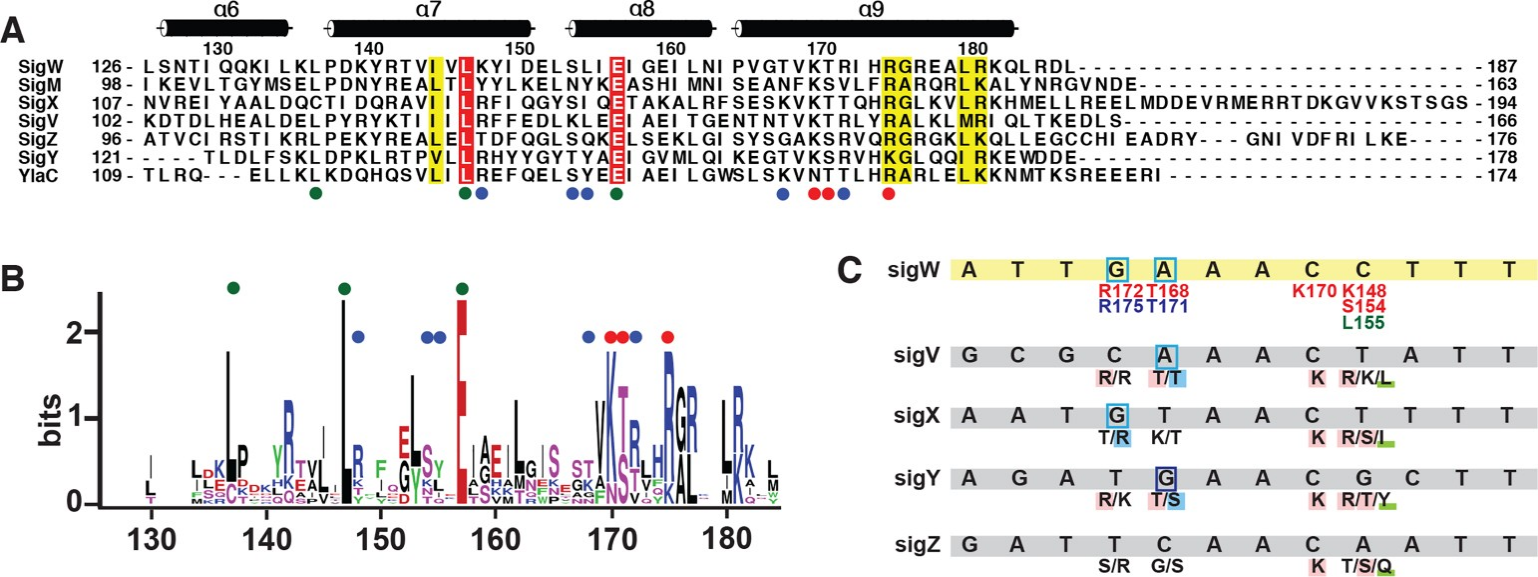
Conserved residues in σ^W^_4_. (A) Sequence alignment of σ_4_ domains of *B*. *subtilis* ECF sigma factors. The highly conserved K137, L148, and E157 are indicated by green circles. Residues that form purine- and backbone-specific hydrogen bonds are indicated by red and blue circles, respectively. (B) Sequence logo for the σ_4_ domains of *B*. *subtilis* ECF sigma factors. (C) The sequences of the -35 promoter elements for *B*. *subtilis* ECF sigma factors. Residues that form hydrogen bonds with the -35 promoter element are predicted based on the interactions between σ^W^_4_ and -35^W^ and the sequence alignment in (A).

*B*. *subtilis* contains multiple ECF sigma factors that respond to diverse environmental stresses. The positions L137, L147, and E157 of σ^W^_4_ are highly conserved in *B*. *subtilis* ECF sigma factors (**Fig 5A and 5B**). The highly conserved residues are likely to be involved in the intrinsic folding of SigW, but not in DNA binding. L137 and L147 stabilize σ^W^_4_ as part of the central innermost hydrophobic cluster (**S6A and S6B Fig**). E157 is associated with σ^W^_2_ binding in the crystal structure of the SigW/RsiW complex (**S6C and S6D Fig**).

The residues involved in DNA binding are less conserved than those involved in intrinsic folding. Residues K170, T171, and R175, which mediate purine-specific hydrogen bonds in σ^W^_4_, are aligned to K/R, S/T, and K/R in *B*. *subtilis* ECF sigma factors, as well as *E*. *coli* SigE (**Fig 5A and 5B**). The conservation of these residues correlates with the three nucleotides that mediate purine-specific interactions (G_-35_, A_-34_, and G’_-31_ in -35^W^). For example, A_-34_ in the SigV promoter interacts with T144 and T147, as does A_-34_ in -35^W^, and T168/T171 in σ^W^_4_, whereas C_-34_ in the SigZ promoter is aligned with G138 and S141. These observations suggest that sequence variation in the DNA-binding interface confers the specificity required to discriminate between -35 elements (**Fig 5C**). In summary, conserved residues in *B*. *subtilis* ECF σ_4_ are mainly involved in intramolecular stability and interactions with the -35 element. Slight sequence variations in the residues involved in the binding of the -35 element may contribute to the fine-tuning of the promoter selectivity and binding affinity for individual ECF sigma factors.

## Conclusions

Group IV ECF sigma factor is activated in response to environmental stress and initiates transcription of its own regulon to counter that stress. The crystal structure of σ^W^_4_/-35^W^ shows that SigW selectively recognizes cognate -35 promoter elements which have a narrowed minor groove, in a similar manner to the *E*. *coli* SigE. Comparison with the SigW/RsiW structure shows that SigW binding to the -35 promoter element and anti-sigma RsiW is mutually exclusive. These results provide the structural basis for the mechanism of SigW activation, and improve our understanding of the selective interactions between σ_4_ domains and their cognate -35 promoter elements.

## Supporting information

S1 Fig Overall electron density map of σ^W^_4_/-35^W^. Red and orange stick models indicate σ^W^_4_ and -35^W^, respectively. 2Fo-Fc maps are drawn at two different orientations. The contour level is set to 1.0 σ.
(TIF)

S2 Fig Map of electron density around the binding interface in the σ^W^_4_/-35^W^ structure. 2Fo-Fc maps are drawn at same scale and orientation as those for models in Fig 2C and arranged in the same order as the panels in Fig 2C. The contour level is set to 1.0 σ.
(TIF)

S3 Fig Hydrophobic interactions between the σ_4_ domain and the -35 promoter element. (A, B) Hydrophobic interactions between σ^W^_4_ and -35^W^ shown at two different orientations. Residues and nucleotides, which are associated with hydrophobic bonds, are drawn as red and orange stick models. Dotted lines indicate hydrophobic interactions. (C, D) 2Fo-Fc electron density maps in (C) and (D) are shown at same scale and orientation as those for models in S2A and S2B Fig, respectively. The contour level is set to 1.0 σ. (E, F) Hydrophobic interactions between σ^E^_4_ and -35^E^. Residues and nucleotides which are associated with hydrophobic bonds are drawn as purple and green stick models.
(TIF)

S4 Fig Structural comparison of σ_4_ domains. (A) Superposition of σ^E^_4_/-35^E^ and σ^W^_4_/-35^W^ structures. N- and C-termini of σ^W^_4_ are labeled. (B) Distribution of root-mean-square values between Cα positions of superimposed σ^E^_4_ and σ^W^_4_ structures. (C) Superposition of σ^W^_4_ domains from the structures of σ^W^_4_/-35^W^ and SigW/RsiW.
(TIF)

S5 Fig Cation-π interaction. (A) The green dotted line indicates the cation-π interaction between R176 of σ^E^_4_ (cyan model) and C_-36_ of 35^E^ (green). The corresponding residue in σ^W^_4_ (R175) and base in -35^W^ (T_-36_) are drawn as a stick model. The distance between the Arg and pyrimidine is labeled.
(TIF)

S6 Fig Conserved residues of σ_4_ domains. The residues that participate in intramolecular interactions with L137 (A), L147 (B), or E157 (C) are drawn as stick models and the residue number is labeled. The helix and loop in σ^W^_4_ are colored pink and light brown. (D) σ^W^_4_ in SigW/RsiW structure (PDB ID: 5WUQ) is superposed onto that of the σ^W^_4_/-35^W^ structure. SigW in SigW/RsiW structure is colored green. E157 in σ^W^_4_ interacts with T71 in σ^W^_2_ and does not participate in DNA binding.
(TIF)
