# Supplementary figures and images for: Structural analysis of the recognition of the -35 promoter element by SigW from Bacillus subtilis
Source: PLoS One. 2019 Aug 28;14(8):e0221666. doi: 10.1371/journal.pone.0221666 (PMC6713349; doi:10.1371/journal.pone.0221666)

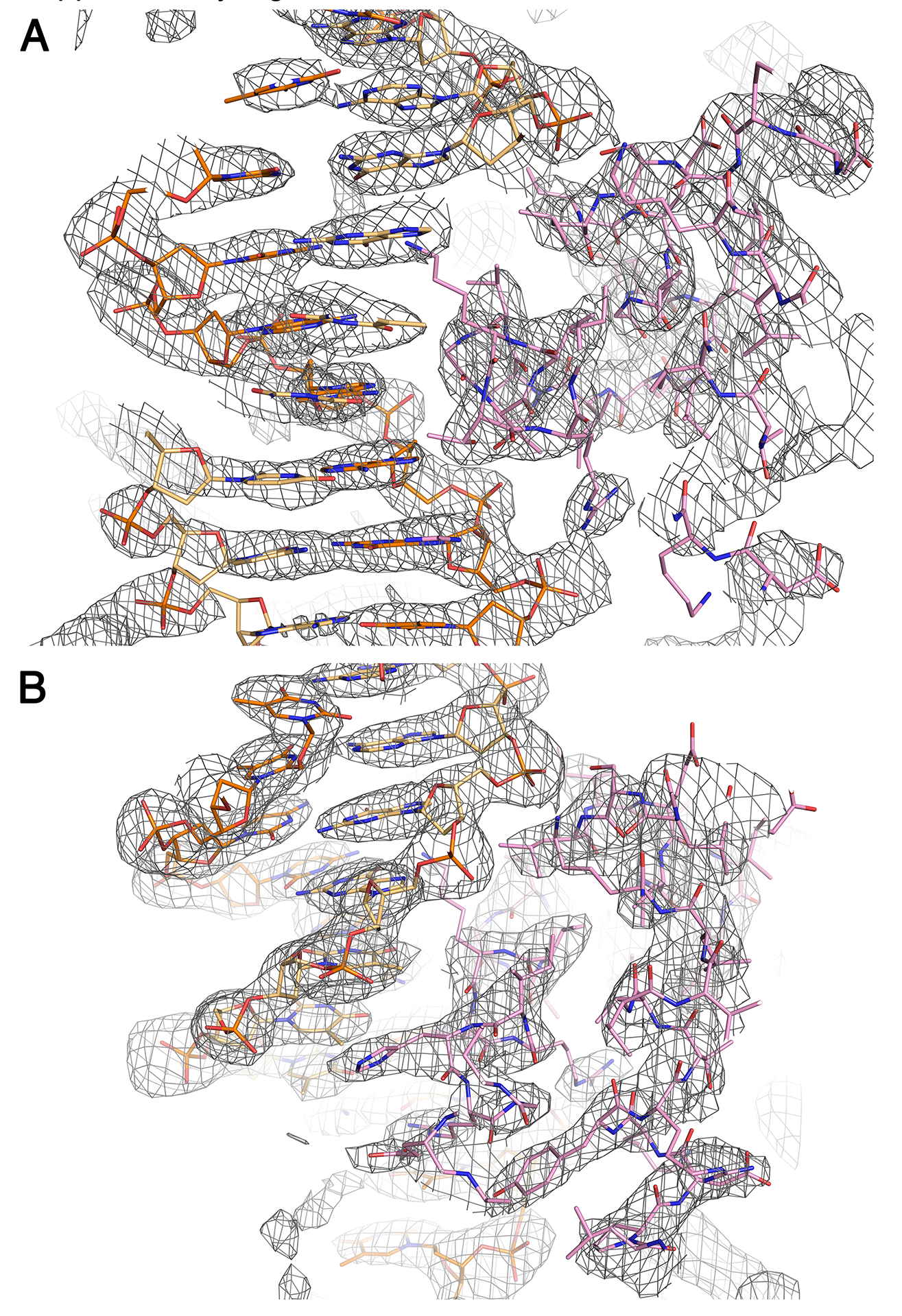

Supplement: S1 Fig — Red and orange stick models indicate σW4 and -35W, respectively. 2Fo-Fc maps are drawn at two different orientations. The contour level is set to 1.0 σ. (TIF) [file pone.0221666.s001.tif]

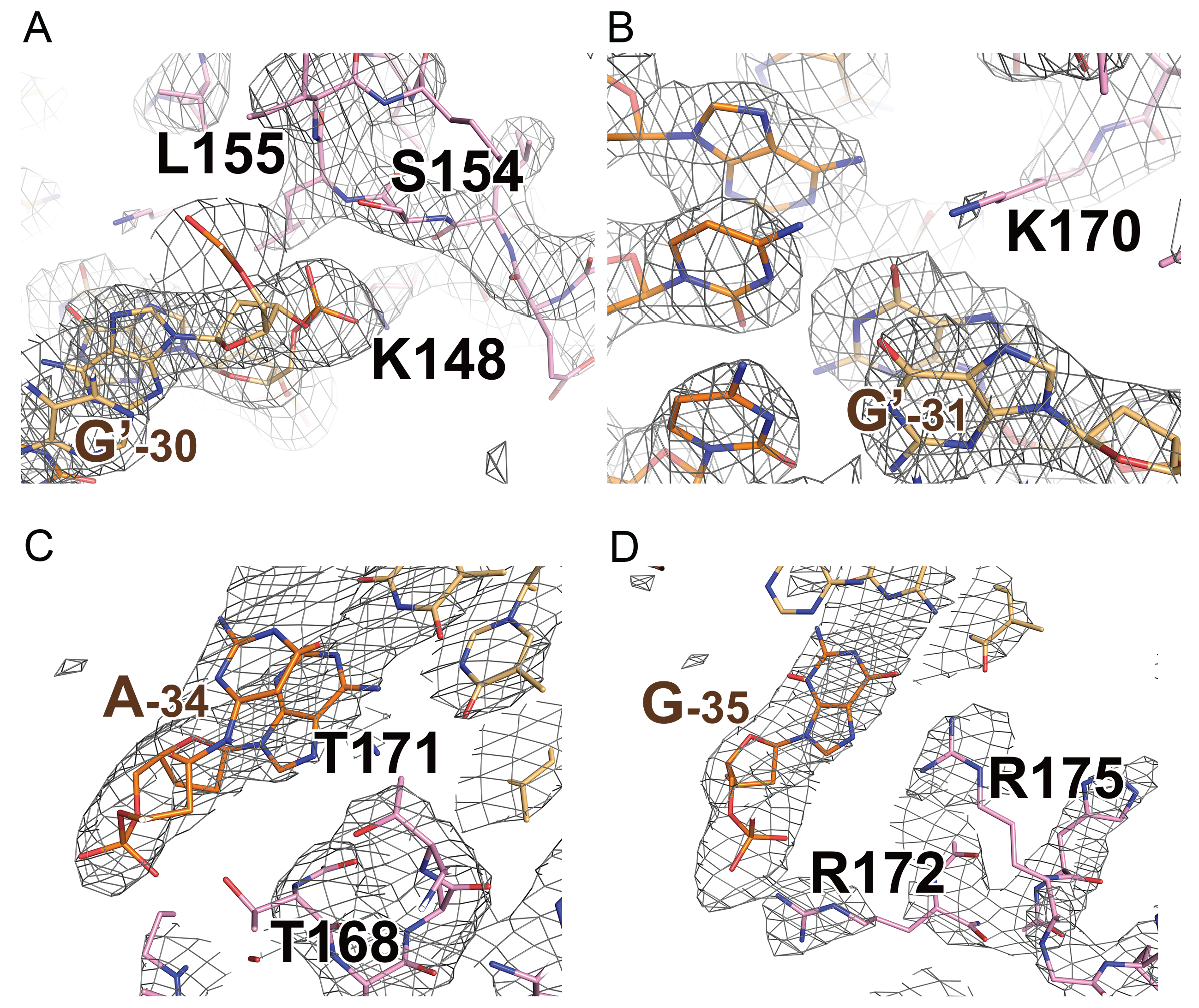

Supplement: S2 Fig — 2Fo-Fc maps are drawn at same scale and orientation as those for models in Fig 2C and arranged in the same order as the panels in Fig 2C. The contour level is set to 1.0 σ. (TIF) [file pone.0221666.s002.tif]

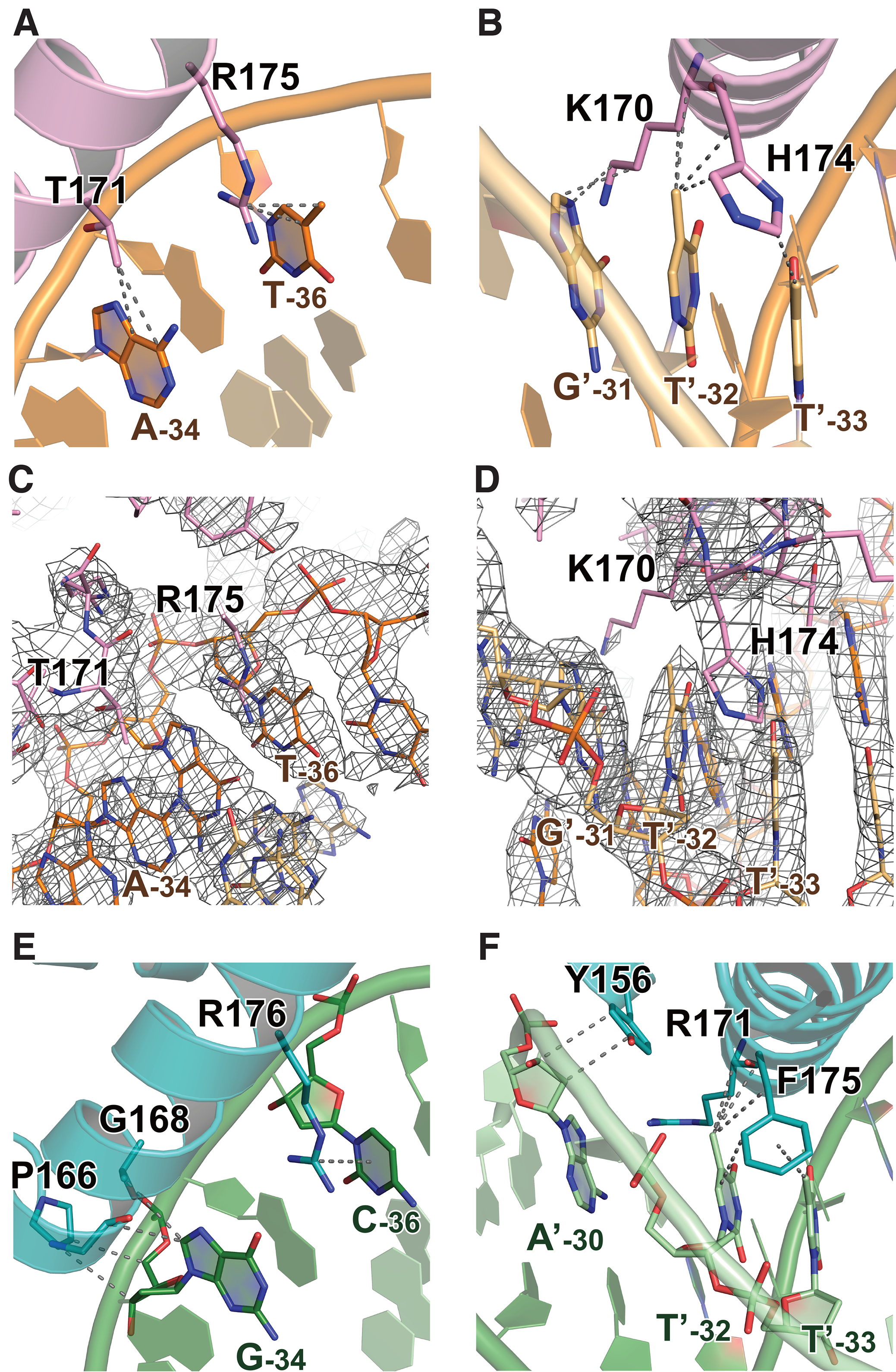

Supplement: S3 Fig — (A, B) Hydrophobic interactions between σW4 and -35W shown at two different orientations. Residues and nucleotides, which are associated with hydrophobic bonds, are drawn as red and orange stick models. Dotted lines indicate hydrophobic interactions. (C, D) 2Fo-Fc electron density maps in (C) and (D) are shown at same scale and orientation as those for models in S2A and S2B Fig, respectively. The contour level is set to 1.0 σ. (E, F) Hydrophobic interactions between σE4 and -35E. Residues and nucleotides which are associated with hydrophobic bonds are drawn as purple and green stick models. (TIF) [file pone.0221666.s003.tif]

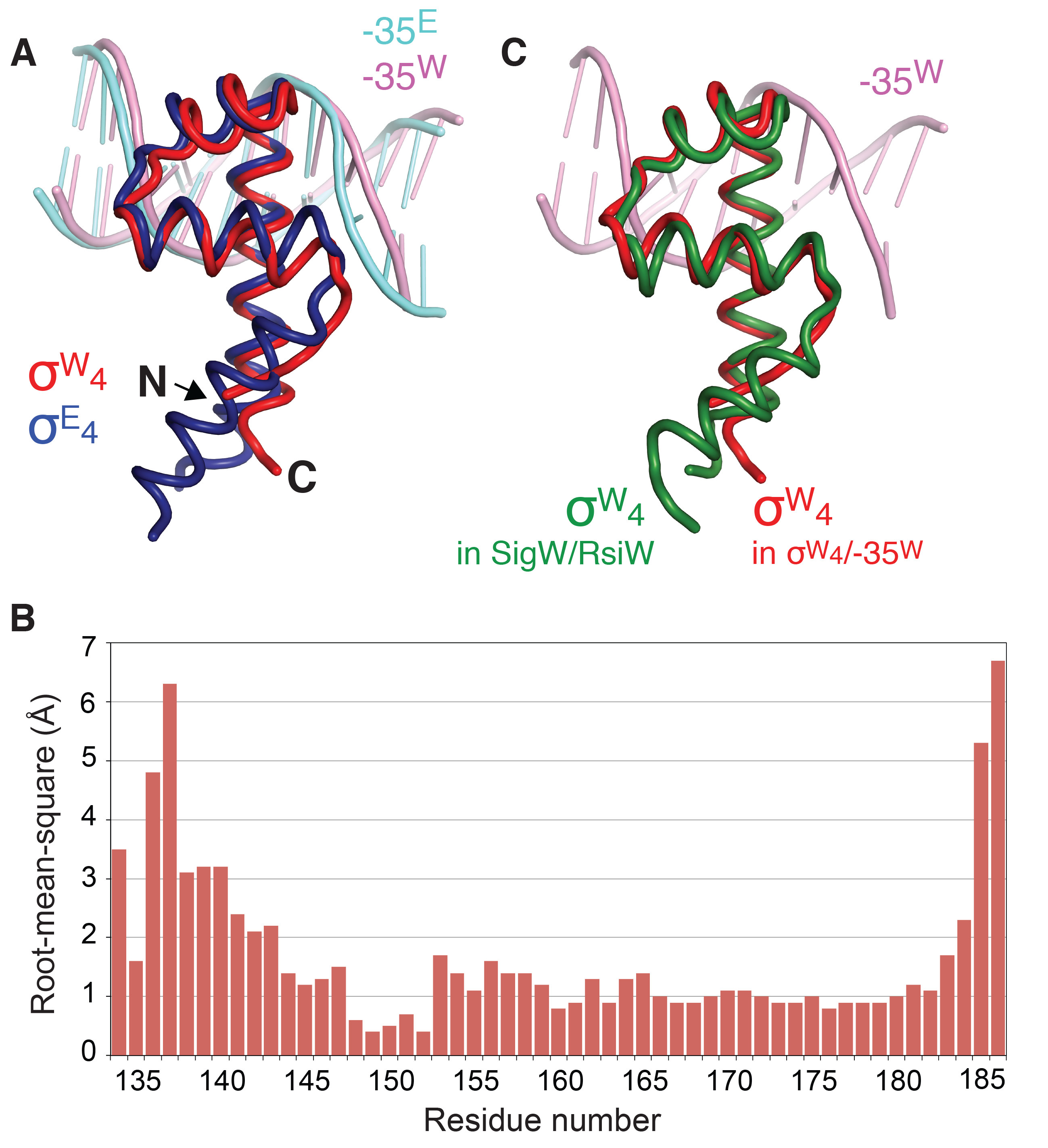

Supplement: S4 Fig — (A) Superposition of σE4/-35E and σW4/-35W structures. N- and C-termini of σW4 are labeled. (B) Distribution of root-mean-square values between Cα positions of superimposed σE4 and σW4 structures. (C) Superposition of σW4 domains from the structures of σW4/-35W and SigW/RsiW. (TIF) [file pone.0221666.s004.tif]

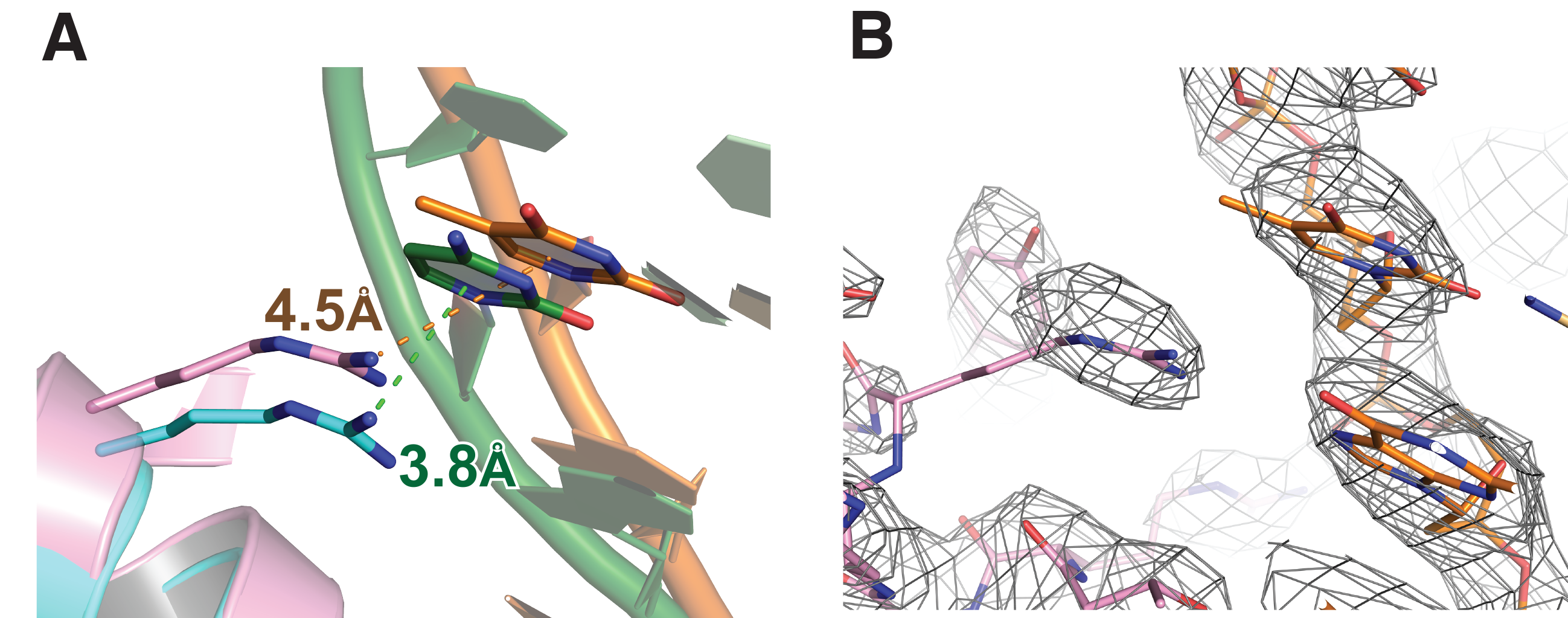

Supplement: S5 Fig — (A) The green dotted line indicates the cation-π interaction between R176 of σE4 (cyan model) and C-36 of 35E (green). The corresponding residue in σW4 (R175) and base in -35W (T-36) are drawn as a stick model. The distance between the Arg and pyrimidine is labeled. (TIF) [file pone.0221666.s005.tif]

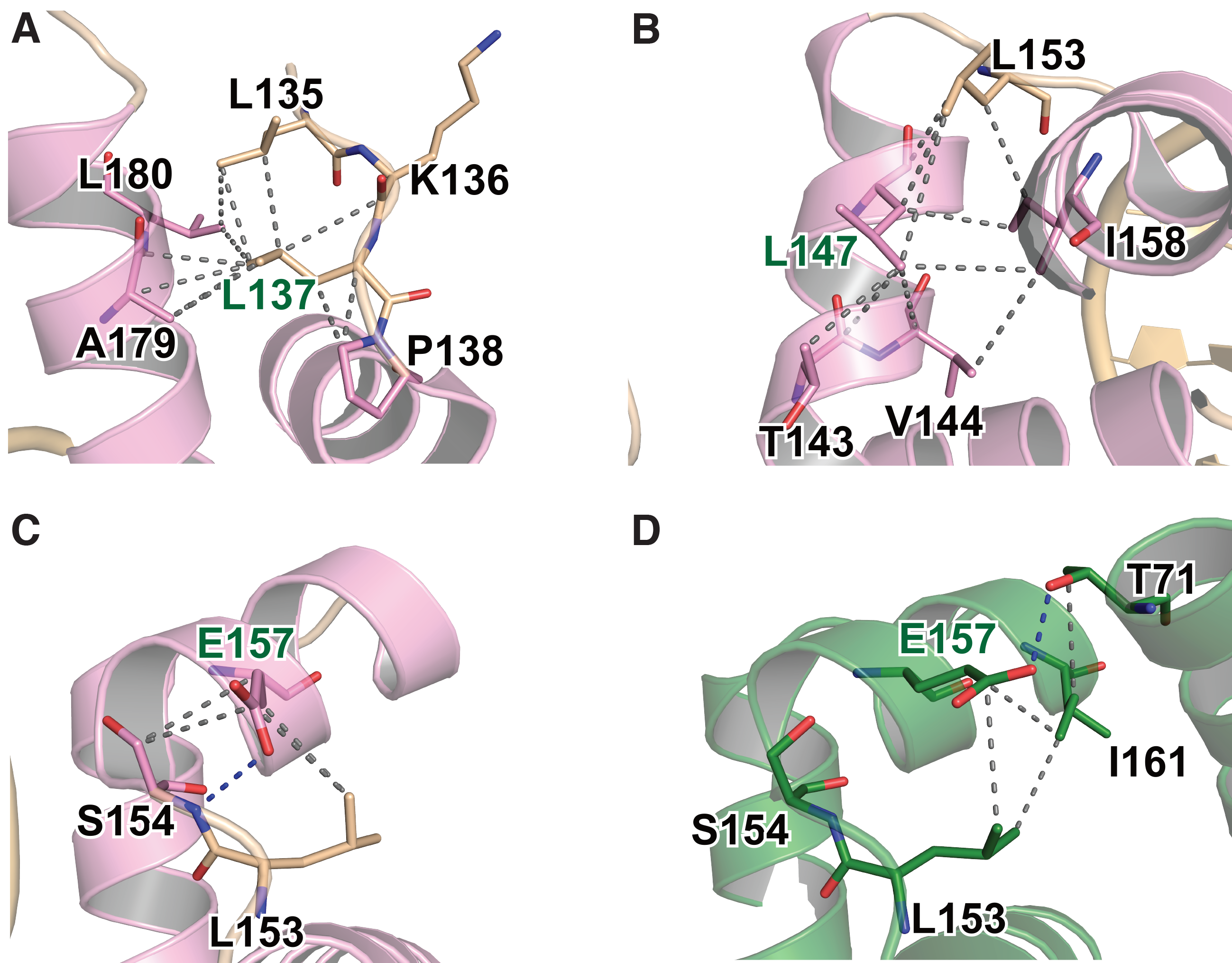

Supplement: S6 Fig — The residues that participate in intramolecular interactions with L137 (A), L147 (B), or E157 (C) are drawn as stick models and the residue number is labeled. The helix and loop in σW4 are colored pink and light brown. (D) σW4 in SigW/RsiW structure (PDB ID: 5WUQ) is superposed onto that of the σW4/-35W structure. SigW in SigW/RsiW structure is colored green. E157 in σW4 interacts with T71 in σW2 and does not participate in DNA binding. (TIF) [file pone.0221666.s006.tif]
